# Optimization of the Zebrafish Larvae Pentylenetetrazol-Induced Seizure Model for the Study of Caffeine and Topiramate Interactions

**DOI:** 10.3390/ijms241612723

**Published:** 2023-08-12

**Authors:** Adrian Bartoszek, Alicja Trzpil, Anna Kozub, Emilia Fornal

**Affiliations:** Department of Bioanalytics, Medical University of Lublin, ul. Jaczewskiego 8b, 20-090 Lublin, Polandemilia.fornal@umlub.pl (E.F.)

**Keywords:** caffeine, topiramate, zebrafish, zebrafish larvae, epilepsy, seizure, PTZ-induced seizure

## Abstract

Epilepsy is a common neurological disorder characterized by seizures that cause neurobiological and behavioral impairment. Caffeine (CAF), which is the most widely consumed stimulant in the world, is reported to influence epileptic seizures and antiepileptic drugs, especially topiramate (TPM). The aim of the study was to optimize the zebrafish larvae pentylenetetrazol-induced seizure model for the study of CAF and TPM interactions, which include the determination of dose space, and the delivery of an analytical method for monitoring CAF, TPM, and CAF metabolite paraxanthine (PAR) in Zebrafish larvae. Methods: The zebrafish larvae, 4 days post-fertilization, were incubated for 18 h with CAF, TPM, or CAF + TPM, with subsequent locomotor activity assessment. Seizures were evoked by adding PTZ solution to obtain a final concentration of 20 mM. Subsequently, the liquid chromatography–mass spectrometry (LC–MS/MS) analytical method was used to simultaneously assess the levels of both CAF and TPM in the larvae. CAF (50 mg/L) and TPM (75 μM) given separately decreased the average larvae locomotor activity compared to the PTZ group but, however, were not able to lower it to the control level. Co-administration of 25 mg/L CAF and 50 μM TPM suppressed the activity to the same level. Adding 25 μM TPM to 50 mg/L CAF decrease the measured CAF level in the larvae. Until proven otherwise, CAF consumption should be regarded as a potential determinant in the modulation of TPM’s efficacy in the management of epileptic seizures. The optimized model will contribute to the standardization of studying CAF and TPM interactions and building the understanding of the molecular bases of the interaction.

## 1. Introduction

Epilepsy is the second most common neurological disorder and is characterized by seizures that cause neurobiological and behavioral impairment [1]. Patients usually have physical (bruises and fractures after a seizure attack) and psychological (anxiety and depression) problems [1]. Unfortunately, despite the availability of more than 30 antiepileptic drugs, there is no definitive cure for the disease with only symptomatic treatment [2], and about a third of the epileptic patient population does not respond to current medications [3].

Caffeine (CAF), which belongs to the group of purine alkaloids, is the most widely consumed stimulant in the world [4]. Its average daily intake in coffee, tea, and soft drinks is about 300 mg per person, which is already a pharmacologically active dose [4]. Moreover, CAF is added to many foods and beverages. Common stimulants containing CAF, such as coffee and tea, affect both epileptic seizures and the effects of anticonvulsant drugs, making seizure control even more difficult [5].

Topiramate (TPM) is a second-generation anticonvulsant drug mainly used for the treatment of epilepsy and migraines [6]. It is approved both as monotherapy and adjunctive therapy. When administered alone, it is characterized by linear pharmacokinetics (PK); however, the clearance is known to be influenced by different factors, including age, renal function, and co-medication [6].

There is little clinical data on the relationship between CAF and epileptic seizures. Most of the current knowledge comes from preclinical animal studies, a few clinical trials, and a few case studies [7,8]. Current data indicate a complex relationship between epileptic seizures, CAF, and antiepileptic drugs, making it impossible to develop clear clinical guidelines for CAF intake among people with epilepsy or people at risk for epilepsy. Preclinical studies indicate that CAF increases susceptibility to epileptic seizures. In some cases, long-term CAF intake may protect against seizures. CAF reduces the efficacy of several antiepileptic drugs, most notably TPM. The relationship between CAF, epileptic seizures, and antiepileptic drugs is complex and not fully understood [7,8].

So far, to the best of our knowledge, there is no study simultaneously assessing the level of CAF and TPM when administered together.

The aim of the study was to optimize the zebrafish larvae pentylenetetrazol (PTZ)-induced seizure model for the study of CAF and TPM interactions. A simple, fast, and sensitive liquid chromatography–mass spectrometry (LC-MS/MS) analytical method was developed and used for the simultaneous determination of CAF, TPM, and PAR in zebrafish larvae. The dose space, where the same reduction in seizures exists, was defined.

## 2. Results

### 2.1. The Influence of Caffeine and Topiramate Combination on Larval Locomotor Activity

**Figure 1:**
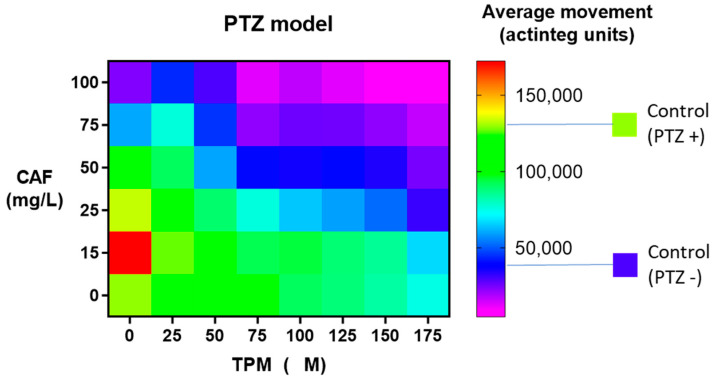
Effect of caffeine (CAF) and topiramate (TPM) combination on average larval locomotor movement in PTZ-induced seizure model; TPM concentration applied: 25, 50, 75, 100, 125, 150, and 175 µM; CAF concentration applied: 15, 25, 50, 75, and 100 mg/L.

Firstly, the average movement of zebrafish larvae, expressed in “actinteg” units, under the influence of CAF (concentration range of 15 to 100 mg/L) and TPM (25 to 175 μM) was assessed in the PTZ-induced seizure model (Figure 1).

When larvae were treated with 25 μM TPM, the addition of 50 mg/L, 75 mg/L, and 100 mg/L of CAF significantly suppressed the activity compared to the PTZ group (*p* < 0.001), and zebrafish treated with 100 mg/L CAF showed activity at the level of control animals. With 50 μM, 75 μM, or 100 μM TPM, 50 mg/L, 75 mg/L, and 100 mg/L CAF suppressed the movement to the control level. With higher TPM doses, 25 mg/L CAF attenuated the activity to that of non-PTZ-treated animals. The combination of TPM in doses 75–175 μM and all investigated CAF dilutions suppressed the movement compared to the PTZ group (*p* < 0.001). The addition of 15 mg/L CAF to TPM did not influence the larval activity, compared to TPM alone, in any investigated TPM dilution, while 50, 75, and 100 mg/L CAF suppressed it in TPM over 25 μM (Figure 1).

**Figure 2:**
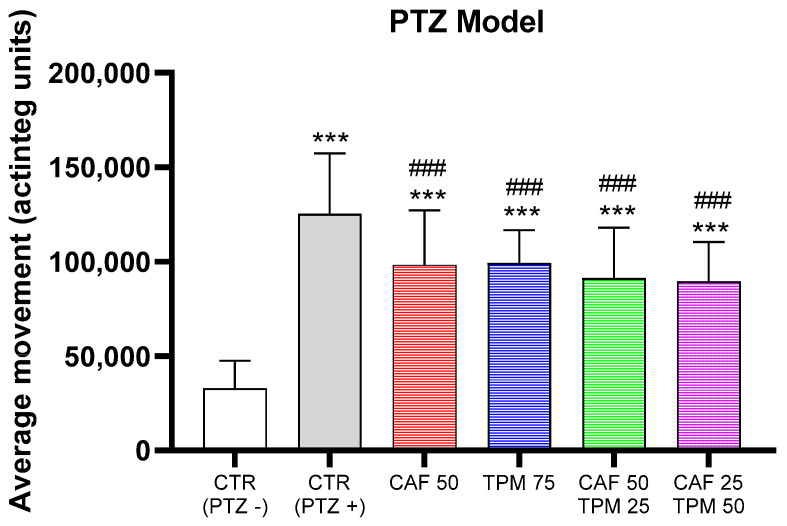
Effect of caffeine (CAF) and topiramate (TPM) combinations on average larval locomotor movement in PTZ-induced seizure model; TPM concentration applied: 25, 50, and 75 µM; CAF concentration applied: 25 and 50 mg/L; *** *p* < 0.001, as compared to control (PTZ−); ### *p* < 0.001 as compared to control (PTZ+).

Based on these results, we have found the concentration of CAF and TPM, alone or in combination, which significantly suppressed the average movement comparing to the PTZ group but did not decrease it to the control level and did not differ between the investigated concentrations (Figure 2).

### 2.2. Caffeine and Paraxanthine Quantification

**Figure 3:**
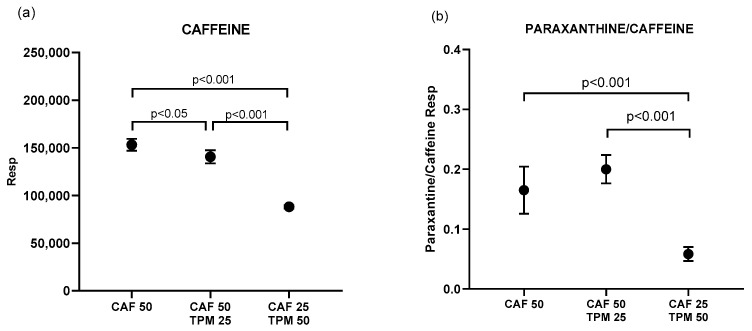
Caffeine (**a**) and paraxanthine/caffeine ratio (**b**) peak area (resp) for the larvae treated with caffeine in PTZ-induced seizure model; caffeine concentration applied: 25 and 50 mg/L.

Subsequently, in order to investigate possible pharmacokinetic interactions, we determined the amount of CAF, expressed as peak area, accumulated in the zebrafish larvae in the above chosen dosages (Figure 3a). Co-administration of TPM (25 μM) with CAF (50 mg/dl) significantly reduced the amount of CAF in the larvae compared to CAF alone in the same dose (50 mg/dl). Zebrafish treated with 25 mg/dl CAF and 50 μM TPM had significantly lower CAF levels than the other investigated conditions (Figure 3a).

We also quantified the primary CAF metabolite—paraxanthine (PAR) (Figure 3b). Unlike CAF, adding 25 μM TPM to 50 mg/dl CAF did not change the PAR/CAF ratio comparing to larvae treated with CAF alone (Figure 3b). Zebrafish treated with 25 mg/dl CAF and 50 μM TPM had significantly lower PAR/CAF ratios than the other investigated conditions (Figure 3b).

### 2.3. Topiramate Quantification

**Figure 4:**
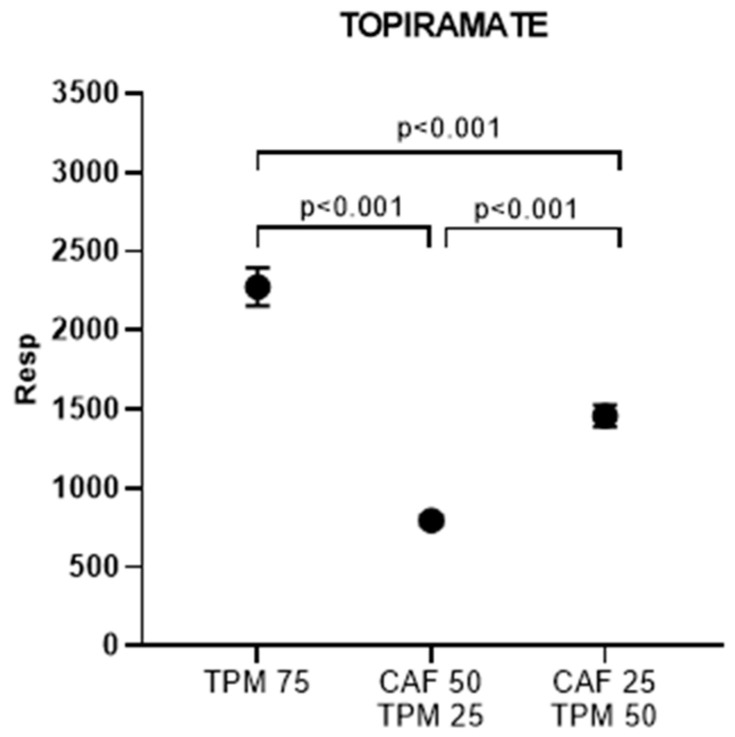
Topiramate peak area (resp) for the larvae treated with caffeine in PTZ-induced seizure model; topiramate concentration applied: 25, 50, 75 µM.

Then, we determined the amount of TPM, expressed as peak area, accumulated in the zebrafish larvae in the above chosen dosages (Figure 4). TPM levels significantly differ between all investigated doses (Figure 4).

## 3. Discussion

We optimized the zebrafish larvae PTZ-induced seizure model for the study of CAF and TPM interactions by determining the CAF and TPM concentration space, resulting in a similar effect in reducing seizure, and developing an analytical method based on ultra-high-performance liquid chromatography followed by mass spectrometric detection to simultaneously quantify CAF, TPM, and PAR in the PTZ-induced seizure model in zebrafish larvae.

### 3.1. The Influence of Caffeine and Topiramate on Larvae Locomotor Activity

Depending on the purpose of the study, different chemical and genetic epilepsy models are described [9]. In both zebrafish and rodents, PTZ-induced seizure is the best-described chemical model yet. The influence of CAF on TPM in chemically induced seizure has already been considered in some animals, but not in zebrafish [8]. In *Danio rerio*, the PTZ model joined the scientific stage in 2005 [9] and was later confirmed in many studies, including those on antiepileptic drugs [10]. Afrikanova et al. presented the anticonvulsant activity of TPM against PTZ-induced seizure (20 mM), which led us to use this model in our study. In a study conducted by Steenbergen et al. 6 dpf larvae were treated with CAF (85 mg/L) for 7 min followed by a rapid washout, which did not alter the total swim distance [11]. In another study carried out in 7 dpf, 4 h treatment (85 mg/L) caused a significant reduction in the traveled distance, and the suppression was more pronounced after 24 h of exposure [12]. Confirming results were observed in 7 dpf larvae exposed for 2 h (acute exposure) prior to analysis and kept in the same solutions during assay [13]. They found that 10 mg/L CAF did not change the swimming speed, while 100 mg/L suppressed it significantly [13]. It was also found that incubation 90 min post fertilization significantly reduces distance at 120 to 168 h post fertilization, especially in dark periods [14].

Our results, regarding suppressed locomotor activity in doses >50 mg/L, are in line with other reports presenting reduced movement: 4 h and 24 h exposure (85 mg/L) in 7 dpf larvae [12]; 2 h exposure (100 mg/L) in 7 dpf [13].

In the study conducted by Zhang et al., after 4 h exposure (4 dpf larvae), TPM was able to slightly increase the total movement in doses of 200 μM, but not 100 μM, and after 24 h, no differences, compared to control, were observed [15]. Incubating 6 dpf larvae for 24 h with TPM did not significantly change the total distance in doses of 1, 3, and 10 mM compared to the control in the non-PTZ group; however, the distance was suppressed in the PTZ model in all three doses [16]. TPM inhibited seizure-like behavior in 7 dpf larvae after 18 h incubation (200 μM) but failed to inhibit total seizure duration in the electroencephalogram assay [10]. In another study, both 24 h incubation and acute exposure to TPM (200 μM) alone induced a significant decrease in locomotor activity in 7 dpf zebrafish [17]. In the PTZ-induced seizure model (10 mM), the total movement for both the TPM pretreatment and the acute exposure groups was significantly lower than the controls. They used a lower PTZ dose (10 mM) for a longer time, as higher PTZ concentrations can manufacture synaptic fatigue, exhaustion, and death, resulting in a reduction in swimming behavior in later time increments [17]. In contrast to Afrikanova et al. who faced discrepancies in locomotor and electroencephalogram assay [10], they found that TPM reduced both behavior and neural activity, assessed with electroencephalogram and GCaMP studies [17].

Our findings suggest that TPM (>50 μM) significantly protected larvae against PTZ but did not suppress the activity to the control level, which is in line with findings among 7 dpf larvae (180 μM, 18 h exposure) [10]. In 7 dpf larvae, 24 h TPM exposure (200 μM) decreased the activity, even to the control level [17].

The number of studies regarding the interaction between CAF and TPM is limited. CAF at doses of 23.1 mg/kg and 46.2 mg/kg, given acutely or chronically, increased the amount of TPM, vital to protecting 50% of the mice (ED50) against maximal electroshock (MES)-induced seizure model [18]. In contrast, CAF in lower doses (5.7 and 11.5 mg/kg) did not affect the anticonvulsant action of TPM. The authors excluded possible pharmacokinetic interaction between tested drugs because, as measured, the CAF did not influence the free plasma concentration of TPM [18]. In rats, CAF at doses 23.1 mg/kg and 46.2 mg/kg significantly enhanced the ED50 value for TPM, when administered either chronically or acutely in MES-induced convulsions [8]. However, in our study, higher CAF doses did not increase the amount of TPM needed to protect the larvae against PTZ, which was observed in both mice [18] and rats [8]. We found that CAF > 25 mg/L decreases the movement to a greater extent than TPM alone. The higher the CAF concentration, the more pronounced the suppression effect that was observed (per unit of TPM concentration).

### 3.2. Caffeine and Topiramate Metabolism

CAF is metabolized in the liver through demethylation, which is catalyzed by the cytochrome P450 enzyme family, almost exclusively CYP1A2 (95%). This enzyme converts CAF to its primary metabolites, including PAR, THM, and THY [19]. PAR is the most abundant (84%) and biologically active metabolite of CAF. It is responsible for most of CAF’s pharmacological effects, including its stimulant properties. The rate of CAF metabolism can vary significantly among individuals, and can be influenced by various factors, such as age, sex, genetics, and environmental factors [20]. The PAR to CAF ratio has been used as a biomarker of CYP1A2 activity, with higher ratios indicating faster CAF metabolism and higher CYP1A2 activity. Measuring this ratio can be useful in predicting individual responses to CAF, as well as in assessing the risk of adverse effects associated with CAF consumption [21].

According to prior studies, the development of the hepatic primordium in zebrafish initiates at 28 hpf, followed by hepatic outgrowth between 60 and 72 hpf, and by 120 hpf, the liver function, which includes CYP metabolism, is nearly fully formed [22]. However, it was shown that CYP metabolic functions (especially CYP1A2) are similar to those of human CYP isoform even before full liver development, as confirmed via both mRNA and metabolite assessment in 24–120 hpf larvae [23].

TPM undergoes limited metabolism and is mainly excreted unchanged through urine, representing roughly 70% of the administered dose. In humans, six metabolites have been identified, with each one individually accounting for less than 5% of the administered dose. It is worth noting that these metabolites of TPM are not recognized to possess any significant activity [24]. TPM is primarily metabolized by enzymes in the liver, including CYP3A4 and CYP2C19, but it is also a weak inhibitor of several other CYP enzymes, including CYP2C9 and CYP2C19. To date, there is no evidence for CYP1A2 influence by TPM [24]. In our study adding 25 μM TPM to 50 mg/dl CAF did not change the PAR/CAF ratio, a biomarker of CYP1A2 activity, compared to larvae treated with CAF alone, which is in line with previous study [24], at least in investigated doses.

Overall, understanding CAF metabolism and the role of CYP1A2 is important for predicting individual responses to CAF and for identifying factors that can affect CAF metabolism, which can ultimately inform strategies for the safe and effective use of CAF-containing products in patients suffering from epilepsy.

### 3.3. Translating Animal Studies into Human

Zebrafish has appeared as an in vivo model during the last two decades and is used at various research stages. Zebrafish larvae are intermediate solutions between in vitro and in vivo mammal studies, because of the model efficiency and the possibility of studying whole vertebrate organisms. They shows 70% genetic homology with humans [25]. Although using larvae for experiments has advantages, it requires an understanding of how the development affects the feature being studied. With the use of paracetamol, it was found that the absorption increased by 106% between 3 and 4 dpf but did not significantly enhance at 5 dpf [26]. On the other hand, drug elimination increased by 17.5% per day in the 3 to 5 dpf period as a consequence of permanent development of enzymatic processes and eliminating organs. *Danio rerio* larvae have lower metabolic rates as poikilotherms, which could contribute to deviations in clearance values, but the correlation to higher vertebrae increases with age, with best fit at 5 dpf [26].

Respecting a non-mammalian model, the central nervous system of *Danio rerio* demonstrates a high degree of homology with humans [27]. It was presented that the dynamics of seizures in zebrafish and humans are remarkably similar, which enhances the transferability of results from the animal to humans [28].

Since most experiments in pharmacology and toxicology are carried out during these early stages, it is crucial to comprehend and measure the impact of development, including how much difference one experimental day can make on the internal exposure of exogenous compounds. One of the possible solutions is to determine the internal concentration of tested compound in the zebrafish larvae which we have presented in the work. A big challenge is also the small size of the larvae, which requires very sensitive methods, which we used in our study.

## 4. Materials and Methods

### 4.1. Animals

*Danio rerio* stocks of the wild type zebrafish strain (AB strain, Experimental Medicine Centre, Medical University of Lublin, Poland) were maintained at a temperature of 26–28.5 °C in a controlled environment (pH ranging between 6.9 and 7.5; conductivity of 550–700; 14/10 h light/dark cycle). Embryos were reared under a standard light/day cycle in an E3 embryo medium in an incubator (IN 110 Memmert GmbH, Buechenbach, Germany). Zebrafish larvae 4 days post-fertilization (dpf) were used for the assays. After the experiment, larvae were immediately killed by immersion in a solution of tricaine (15 μM). All experiments were conducted in accordance with the National Institute of Health Guidelines for the Care and Use of Laboratory Animals and the European Community Council Directive for the Care and Use of Laboratory Animals of 22 September 2010 (2010/63/EU). For the experiment with larvae up to 5 dpf, agreement with the Local Ethical Commission is not required.

### 4.2. Chemicals

TPM, CAF, and PTZ were purchased from Sigma-Aldrich (Saint Louis, MI, USA). All compounds were dissolved in deionized water and diluted in E3 embryo medium (pH 7.1–7.3; 17.4 μM NaCl, 0.21 μM KCl, 0.12 μM MgSO_4_, and 0.18 μM Ca(NO_3_)_2_) to achieve a designated concentration.

### 4.3. Evaluation of Locomotor Behavior

Larvae were preincubated in 100 mL of E3 embryo medium or tested substances for 18 h in individual wells of a 96-well plate at 28 °C. A total of 10 larvae were used per treatment parameter and per experiment. After the preincubation, 100 mL of E3 embryo medium or 100 mL of a 40 mM PTZ solution were added to obtain a final concentration of 20 mM in order to evoke seizures [10]. Larvae were allowed to habituate for 5 min in a dark chamber of an automated tracking device (ZebraBox^TM^ apparatus; Viewpoint, Lyon, France). The total locomotor activity was then quantified using ZebraLab^TM^ software (Viewpoint, Lyon, France) [10]. Average movement or activity was expressed in “actinteg” units. The actinteg value of the ZebraLab^TM^ software is defined as the sum of all image pixel changes detected during the time slice defined for the experiment (30 min). All tracking experiments were performed in triplicate.

### 4.4. Treatments for the Assessment of Caffeine and Topiramate Concentrations

Larvae were preincubated in 500 μL of E3 embryo medium or tested substances for 18 h in a 48-well plate (5 larvae per plate) at 28 °C. The concentrations of CAF and TPM used in the study were chosen based on the evaluation of locomotor behavior. After the preincubation, 250 μL of E3 embryo medium or 250 μL of a 60 mM PTZ solution were added to obtain a final concentration of 20 mM to evoke seizures. Larvae were allowed to incubate for 30 min with PTZ.

### 4.5. Sample Preparation

Larvae were washed with E3 embryo medium three times, placed in a tube (20 larvae per sample), and homogenized via sonication (4 × 5 s) in 150 μL 100 mM NH_4_HCO_3_. Then, samples were incubated on ice for 15 min and centrifuged (15,000× *g*, 15 min, 4 °C). A volume of 100 μL of supernatant was mixed with methanol: ethanol solution (1:1) to obtain a 1:3 ratio, respectively, and vortexed for 30 s. After 15 min incubation in 20 °C and centrifugation (15,000× *g*, 10 min, 4 °C), 250 μL of supernatant was placed in a chromatography vial.

### 4.6. LC-QQQ-MS Analysis

Chromatographic separation was conducted using the Agilent 1290 Infinity II LC system coupled to the Agilent 6470 Triple Quad tandem mass spectrometer equipped with electrospray ionization source Jet Stream Technology (Agilent Technologies, Santa Clara, CA, USA). Analyte determination was performed using dynamic multiple reaction monitoring (dMRM) in positive ionization mode. The chromatographic separations were conducted using an RRHD Zorbax Eclipse Plus C18 column (Agilent Technologies; 2.1 × 50 mm; 1.8 μm) at a flow rate of 0.3 mL/min. The mobile phases were 0.1% formic acid in water (A) and 0.1% formic acid in methanol (B). The run time was 7 min, with the gradient program 0–4 min, 13%–18% B; 4.01–6 min, 60%–75% B; 6.01–7 min, 95% B, column conditioning 3 min. The injection volume was 2 μL and the column temperature was maintained at 40 °C. The mass spectrometer was operated in positive electrospray ionization mode (ESI+) with the following settings: ion source gas, N2; ion source gas temperature, 300 °C; ion source gas flow rate, 12 L/min; nebulizer pressure, 40 psi; sheath gas temperature, 350 °C; sheath gas flow rate, 12 L/min; and capillary voltage, 4000 V. After each injection, the sampling needle was washed with mixture of methanol and water (80:20, *v/v*). The MS parameters, such as product ion, fragmentor, and collision energy, were optimized and provided in Table 1. The data were acquired using Mass Hunter Acquisition B.09.00 software and processed using Mass Hunter Quantitative 10.02 software (Agilent Technologies, Santa Clara, CA, USA).

### 4.7. Statistical Analysis

Statistical analyses were performed in GraphPad Prism 8 (GraphPad Software, San Diego, CA, USA). For comparison, analysis of variance was performed (one-way or two-way ANOVA). One-way ANOVA was followed by Tukey’s test (post hoc test). In the case of two-way ANOVA, Bonferroni’s test was used as a post hoc test. The confidence limit of *p* < 0.05 was considered statistically significant. Data are presented as mean ± standard deviation (SD). Zebrafish larvae were randomly allocated to experimental groups. The experiments were performed in triplicate.

### 4.8. Study Limitations

The above-discussed study shows contradictory results, which may arise from the use of different models referring to different epilepsy types, various ways of inducing seizures, various methods for drug administration, different doses of chemical substances, and alternative measurement devices, acquiring methods, and assessed parameters.

### 4.9. Study Impact

The optimized model will contribute to the standardization of studying CAF and TPM interactions and building the understanding of molecular bases of the interaction. The findings strengthen the previous research results that CAF is a factor in epilepsy treatment. Until proven otherwise, CAF consumption should be regarded as a potential determinant in the modulation of TPM’s efficacy in the management of epileptic seizures.

## 5. Conclusions

Taking everything into account, we optimized the zebrafish larvae PTZ-induced seizure model for the study of CAF and TPM interactions with an analytical method based on ultra-high-performance liquid chromatography followed by mass spectrometric detection to simultaneously quantify CAF and TPM in the PTZ-induced seizure model in zebrafish larvae. The method can be used for future investigations assessing complex and not fully understood CAF and TPM interactions. It can be also used for monitoring the internal concentration of tested compounds in zebrafish larvae, since internal accumulation, rather than exogenous concentration dissolved in medium, derives pharmacological effect. Most previous studies assessed the concentration dissolved in medium rather than in larvae, which can lead to incorrect interpretation of observed outcomes.

Additionally, we have marked out the area of different CAF and TPM doses which exert the same effect on locomotor behavior in the PTZ-induced seizure model in zebrafish larvae, which could be the base for future metabolomic and proteomic studies. Investigating metabolic processes in zebrafish presents an exciting opportunity to explore such processes in epilepsy and potentially discover new biomarkers for controlling the diseases and create guidelines for caffeine intake.

As previously suggested, until proven otherwise, it should be considered as one factor that CAF consumption may help to maintain control of epileptic seizures [8].

## Figures and Tables

**Table 1:** MRM parameters for caffeine and topiramate.

Analyte	Formula	Retention Time (min)	Retention Window (min)	Precursor Ion *m/z*	Product Ion *m/z*	Fragmentor (V)	Collision Energy (V)
Caffeine	C_8_H_10_N_4_O_2_	5.29	0.5	195.1	110	136	25
				195.1	138	136	21
				195.1	83	136	29
				195.1	69.1	136	33
				195.1	56.1	136	37
Paraxanthine	C_7_H_8_N_4_O_2_	3.33	0.5	181.1	124	118	22
				181.1	96	118	26
				181.1	69.1	118	38
				181.1	67	118	42
				181.1	55.1	118	34
Topiramate	C_12_H_21_NO_8_S	5.83	0.5	340.1	184	136	13
				340.1	264	136	5

## Data Availability

Not applicable.
